# Supplementary material for: Splice-Junction-Based Mapping of Alternative Isoforms in the Human Proteome
Source: Cell Rep. Author manuscript; Available in PMC 2020 Jan 15. (PMC6961840; doi:10.1016/j.celrep.2019.11.026)
Supplement: 2 [file NIHMS1546469-supplement-2.zip › DF1/PXD006675/RightVentricle/RightVentricle_14_MYBPC3_RRTDSHEDTGILDFSSLLK.pdf]

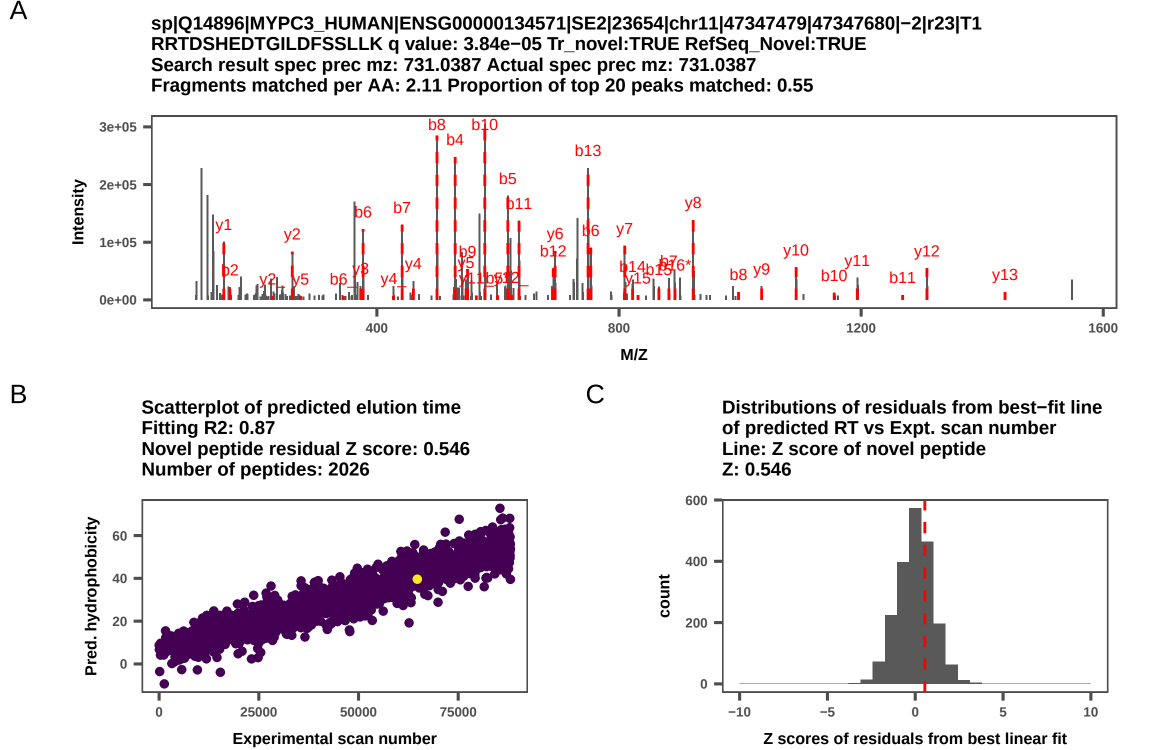
A
sp|Q14896|MYPC3_HUMAN|ENSG00000134571|SE2|23654|chr11|47347479|47347680|−2|r23|T1
RRTDSHEDTGILDFSSLLK q value: 3.84e−05 Tr_novel:TRUE RefSeq_Novel:TRUE
Search result spec prec mz: 731.0387 Actual spec prec mz: 731.0387
Fragments matched per AA: 2.11 Proportion of top 20 peaks matched: 0.55
Intensity
3e+05
2e+05
1e+05
0e+00
b8
b10
b4
b13
b5
b11
y8
b6
b7
y1
y2
y6
b6
y7
b12
b9
y10
y12
y11
y13
b2
y2
y5
b6
y3
y4
y5
b14
b15
b7
b16*
b8
y9
b10
b11
y15
400
800
1200
1600
M/Z
B
Scatterplot of predicted elution time
Fitting R2: 0.87
Novel peptide residual Z score: 0.546
Number of peptides: 2026
Pred. hydrophobicity
60
40
20
0
0
25000
50000
75000
Experimental scan number
C
Distributions of residuals from best−fit line
of predicted RT vs Expt. scan number
Line: Z score of novel peptide
Z: 0.546
count
600
400
200
0
−10
−5
0
5
10
Z scores of residuals from best linear fit
